# Can virtual reality balance games enhance activities of daily living among stroke survivors?

**DOI:** 10.1186/1471-2458-14-S1-P19

**Published:** 2014-01-29

**Authors:** Devinder Kaur Ajit Singh, Nor Azlin Mohd Nordin, Noor Azah Aziz, Siti Norfadilah Abu Zarim, Lim Beng Kooi, Soh Li Ching

**Affiliations:** 1 Faculty of Health Sciences, University Kebangsaan Malaysia, Jalan Raja Muda Abdul Aziz, 50300 Kuala Lumpur, Malaysia; 2 Faculty of Medicine, University Kebangsaan Malaysia, Jalan Raja Muda Abdul Aziz, 50300 Kuala Lumpur, Malaysia; 3 Department of Medical Rehabilitation, University Kebangsaan Malaysia, Jalan Raja Muda Abdul Aziz, 50300 Kuala Lumpur, Malaysia

## Background

Stroke survivors with residual physical disabilities are reported to have decreased muscle strength and balance. Balance ability is one of the prerequisite for performing activities of daily living independently. Virtual reality balance games performed in the community based rehabilitation settings may be beneficial for stroke survivors to improve their functional balance resulting in increased independence in activities of daily living. The aim of this study was to evaluate the use of virtual reality balance games in improving activities of daily living among stroke survivors.

## Materials and methods

This pre-test and post-test study involved 15 and 13 participants in the experimental and control group, respectively from two stroke community-based rehabilitation centres. Experimental group had 1.5 hour of standard physiotherapy exercises with an added half an hour of virtual reality balance games as an intervention. The control group continued their 2 hours of standard physiotherapy exercises. Interventions were performed for 6 weeks in a twice a week session. Barthel index was used to evaluate activities of daily living for pre and post intervention.

## Results

The results showed significant main effect of group on Barthel index score, F(1, 25) =5.53, p=0.04.

## Conclusions

These results suggested that virtual reality balance games as an added activity to standard physiotherapy compared to standard physiotherapy alone was beneficial in enhancing activities of daily living among community dwelling stroke survivors.

